# Intention to use condom among students in Agena preparatory school, Guraghe Zone, Ethiopia: with the application of health believe model

**DOI:** 10.1186/0778-7367-71-23

**Published:** 2013-09-08

**Authors:** Getachew Gebreselassie, Negussie Deyessa, Gezahegn Tesfaye

**Affiliations:** 1 Health System Strengthening Department, Management Science for Health, Addis Ababa, Ethiopia; 2 School of Public Health, Addis Ababa University, Addis Ababa, Ethiopia; 3 Department of Public Health, College of Health and Medical Sciences, Haramaya University, Harar, Ethiopia

**Keywords:** Condom, Intention to use, Health believe model, Students

## Abstract

**Background:**

HIV/AIDS is affecting the majority of the population, particularly the productive age group between 15–49 years resulting in social and economic crisis. The rate of HIV infection would undoubtedly be lowered if safe sexual practices such as correct and consistent use of condoms had been followed. The aim of this study was therefore to assess intention to use condom among students in Agena preparatory school, Guraghe zone, Ethiopia. Agena is an urban area in south Ethiopia.

**Methods:**

Institution based cross-sectional study was conducted. A two stage sampling was applied by stratifying students in to (grade 11 and 12) with each grade having four section (A, B, C, D). Then systematic random sampling was used to select students in each section. Analyses of frequencies and summary measures like mean and Standard Deviation of selected variables were done. Bivariate and multivariate analysis was done to measure the association between different variables.

**Results:**

Out of 450 respondents 122(27.1%) had history of sexual intercourse. Of whom the majority 86(70.5%) had two or more sexual partners. And 45(37%) never used condom, 12(9.8%) used condom sometimes and 65(53.2%) used condom every time during sex. About 300(67.7%) of the respondents have no intention to use condom in the next sexual encounter. On multivariate analysis those students who have high perceived susceptibility (AOR = 1.94 (1.16-3.2)) and high self-efficacy (AOR = 27 (14.4-54.2)) were more likely to have intention to use condom than others.

**Conclusions:**

Intention to use condom in the next sexual intercourse is very low. Information Education and Communication (IEC) on reducing number of sexual partners along with condom use promotion targeting in-school adolescents should be the primary strategy of HIV/AIDS prevention process.

## Background

Although AIDS remains one of the world’s most serious health challenges, global solidarity in the AIDS response during the past decade continues to generate extraordinary health gains. While much of the news on AIDS is encouraging, challenges remain. Globally 34.0 million [31.4 million–35.9 million] people were living with HIV at the end of 2011. An estimated 0.8% of adults aged 15–49 years worldwide are living with HIV. Sub-Saharan Africa remains most severely affected with nearly 1 in every 20 adults (4.9%) living with HIV and accounting for 69% of the people living with HIV worldwide [1].

The routes of transmission of HIV are the same around the world but differing patterns of human behaviour causes the HIV virus to transmit more in certain social networks. In sub-Saharan African countries more than 80% of the mode of transmission of HIV/AIDS is heterosexual intercourse. Over 90% of infections in children resulted from mother to child while women contribute for 55% of all HIV–positive adults [2]. Despite this alarming figure, AIDS is still blighting the lives of another 16,000 people worldwide everyday out of which 50% of them are among young aged 15–24 years [3].

The rate of HIV infection would undoubtedly be lowered if safe sexual practices such as correct and consistent use of condoms had been followed. But, many African men and women find condoms incompatible with their sexual and cultural norms. Moreover, those who would like to use condoms often find them inaccessible and /or unavailable. And, even when available, both male and female frequently shy away from taking advantage of them, feeling that they will be stigmatized. On the other hand in most African countries male condom that has practical usefulness to protect against HIV and other sexually transmitted infections (STIs) has limitations such as religious disapproval, perceived reduced sexual satisfaction, distribution and storage problems which affect the adolescents intention to use condom [4].

The social stigma associated with HIV/AIDS, the disease’s long period of invisibility and the determination of whether the infection is related to behavioural risks such as sexual transmission or occupational exposure add to the complexity of HIV/AIDS in many countries especially the Sub-Saharan Africa [5].

Behaviour change programmes seek to promote safer individual behaviour as well as changes in social norms that generate healthier patterns of sexual behaviour. HIV behaviour change programmes have largely been measured against the outcomes of reduction in the number of young people initiating sexual intercourse early and the number of sexual partners and increase in the correct and consistent use of condoms among people who are sexually active [1].

Generally, no intervention aimed at changing behaviours to promote health can be 100% effective. Because some of the behaviours and activities that need to change behaviour in order to avert HIV Infection are pleasurable, it should be no surprise if short term interventions did not lead to immediate and permanent behavioural change. By any means, there is no question that the prevention programs through behavioural change works and remains the best and most cost-effective approach [6].

Ethiopia is one of the seriously affected countries by the epidemic. The disease is affecting the majority of the population, particularly the productive age group between 15–49 years, resulting in social and economic crisis. Though continuous IEC interventions have made efforts in increasing awareness about modes of transmission and prevention of HIV/AIDS; they have not successfully been able to bring about the desired behavioural change among the population.

Some studies among students in Ethiopia showed high rate of sexual experience and low tendency to use condom. A study in Gonder College of health sciences revealed that out of 383 students 56.1% were sexually active. Among the sexually active students, 37.1% reported ever use of condom. Consistent condom use was reported only by 6.4% [7]. Also a study in Agaro high school showed that 90(25%) of the students had history of sexual intercourse. Among those who had previous sexual exposure 49(54.4%) used condom at least once. Of these, 23(46.9%) were using condom always [8].

In Ethiopia there is lack of extensive research on factors that influence their intention to use condom that use behavioural change models to inform health programmers and policy makers to refine the existing HIV/AIDS prevention and control strategy among youths. Since the health believe model is a behavioural change framework which can addresses several constructs influencing health behaviours such as condom use, it is anticipated that the results of this study can provide a basis for designing an effective HIV/AIDS prevention and control programme in-school youths in Ethiopia.

More over the study area is characterized by high population growth, density and migration of men to bigger towns. As a result, HIV/AIDS infection is estimated to be high. Research on socio-demographic and psychosocial determinants of condom use particularly in the area is scarce (non-existent). Thus, this study is believed to fill the existing information gap. Therefore the aim of the study was to assess intention to use condom among students in Agena preparatory school, Guraghe zone, Ethiopia.

## Methods

### Study setting

The study was conducted in Agena preparatory school, Ezza woreda, Guraghe zone, Ethiopia. Guraghe is one of the diverse ethnic groups found in Southern Nation Nationalities and Peoples Region (SNNPR) in Ethiopia and administratively one of the zones in SNNPR. In Ezza woreda there were four high schools, one preparatory school and one TVET (Technical and Vocational Educational Training) college. The woreda is located 198 Km away from Addis Ababa in the Southern part of Ethiopia. It is one of the 13 woredas of Guraghe zone having 28 Kebeles from which one is urban (Agena) and the rest are rural. The study was conducted from December, 2010 to January, 2011.

### Study design

Institution based cross-sectional study design was used to conduct the study.

### Source population

All high school students of Agena town who enrolled for the academic year of 2010/2011.

### Study population

Preparatory school students enrolled for academic year of 2010/2011. Those students who were blind, critically sick (to the extent of unable to read and write), not voluntary to participate and absent from class at the time of data collection were excluded from the study.

### Sample size determination

The sample size was determined using single population formula by taking the following assumptions; proportion of students (49%) who intend to use condom in Agaro high school [8], marginal error (0.05), Z score of 95% CI Zα/2 = 1.96 and non response rate of 20%. Therefore the total sample size for this study was 460 students.

### Sampling procedure

A two stage sampling method was used to select study participants from the school. The first stage was to stratify students into two categories, grade 11 and 12.With in each grades there were four sections labelled as A, B, C and D. The number of study subjects from each grade was allocated proportionally to the size of the respective sections. The second stage of sampling was to select students with in each sections using systematic random sampling. For the systematic random sampling list of students in each section was used as a sampling frame. By calculating the sample interval, the first person was selected using lottery method and subsequent students were selected by adding the calculated sampling interval for each section.

### Data collection method and tools

A structured self-administered questionnaire was adapted from the standardized Behavioural Surveillance Survey questionnaire [9]. Additionally Health Believe Model (HBM) was used as a conceptual framework for the development of the instrument. Two to six items were derived and used from HBM to measure perceived susceptibility/severity, perceived benefits, perceived barriers and self efficacy using a scale of 5 for “strongly agree” all the way down to 1 “strongly disagree”. The questionnaire was initially prepared in English and then translated in to Amharic and checked for any inconsistencies or distortions in the meaning of words and concepts. Self administered questionnaire using paper and pencil was used for data collection. Five health professionals who can speak both Guraghe and Amharic language and familiar with local customs were recruited to facilitate the overall data collection. They were trained for three days on procedures and techniques of collecting the data.

### Data quality assurance

Proper designing of data collection instruments was made by adopting it from a standardized behavioural surveillance survey. Strict supervision of data collection process was done and training was given to data collectors. Two round pre-test was conducted, the first was focused test among 10–12 students and the other was carried out on similar setting but out of the study area on about 20–30 students. An appropriate modification was made after discussing with the coordinators before starting the actual data collection process.

### Data entry and analysis

Data was entered into Epi info version 3.5.1 and analyzed using SPSS version 16 computer software packages. Data cleaning was carried out using simple frequency and tabulation to look for consistency. Dummy tables that consider the main research questions were drafted. Analysis of frequencies of different variables and chi square test for some selected variables were done. Odds ratios were calculated to determine the strength of association between selected variables. Multivariate analysis using logistic regression was done to control the effect of each explanatory variable on the outcome variable.

Health Belief Model theory constructs was applied in the analysis. It is a psychological model that attempts to explain and predict health behaviour by focusing on attitudes and belief of an individual. The key variables of health belief model used were perceived susceptibility, perceived benefit, perceived severity, perceived barrier and self efficacy.

### Study variables

#### Dependent variables

Intention for condom use: - Regardless of their past sexual experience respondents were assessed for their intention to use condom in the next sexual encounter using the following item;

“I intend to use condom at the next sexual intercourse” Responses were arranged from strongly agree to strongly disagree in 5 scale.

### Independent Variables

Socio-demographic characteristics, perceived susceptibility (severity), perceived benefits, perceived barriers and self-efficacy.

Perceived susceptibility; students were asked four questions regarding perceived susceptibility to HIV/AIDS. The item includes “I'm at low risk for HIV infection”, “I'm too young to get an HIV infection”.

Perceived severity; students were asked three questions regarding perceived severity of the HIV/AIDS virus. The questions included, “if I had an HIV infection, my family relationships would be strained” and “if I got AIDS, I would eventually die from it”.

Perceived benefits; consisted of two items that suggest among other things that, condom is an effective way of preventing the transmission of the AIDS virus.

Perceived barriers; consisted six items, which among others suggests that buying or using condom is embarrassing, expensive, and indicates mistrust.”

Self-efficacy; two items were used to assess the self-efficacy. The items like “confidence to using condom in the middle of sexual excitement”, “high confidence in using condom indifferent situation like after alcohol use” were included.

For the above psychosocial variables a sum score was constructed by adding the items corresponding to each variable and dichotomized in to low and high. The variables score less than or equal to the mean score were considered low where as those greater than the mean score were high. The response options for each item in the variables were on a 5 point likert scale ranging from “strongly agree to strongly disagree”.

### Ethical consideration

Ethical clearance was secured from institutional review board of University of Gondar and ACIPH. Based on the ethical clearance, official permission was obtained from different authorities of the Guraghe zone. The respondents were informed about the objective and purpose of the study. Oral consent was obtained from each respondent before administering the questionnaire. To assure confidentiality no name or personal identifying information was written on the questionnaire and information was recorded anonymously.

## Results

### Socio-demographic characteristics

Four hundred fifty students responded and filled the questionnaire from the sampled 460 students making a response rate of 98%. Majority 278 (61.8%) of the respondents were males. The average age of the study population was 17.93 years. Four hundred forty (97.8%) of the respondents were Guraghes and 10(2.2%) were Amharas. Orthodox Christian constituted 316(70.2%) of the study population. Most of the respondents, 449 (99.8%) were not married at all, the rest insignificant number 1(0.2%) were married which was omitted from subsequent further analysis. Regarding the level of education, 234 (52.0%) were grade 12 students while grade 11 students constituted 216 (48%) of the study population (Table 1).

**Table 1:** Socio-demographic characteristics of students in Agena preparatory school, Guraghe zone, Ethiopia, 2011

**Variables**	**Frequency**	**Percent (%)**
Sex		
Male	278	61.8
Female	172	38.2
Age group		
15–19	410	91.1
20–25	40	8.9
Religion (n = 449)		
Orthodox Christian	316	70.2
Muslim	115	25.6
Protestant	18	4
Ethnicity		
Guraghe	440	97.8
Amhara	10	2.2
Marital status (n = 449)		
Single	449	99.8
Educational status		
Grade 11	216	48
Grade 12	234	52
Monthly family income		
100–500 birr ($5-26)	145	32.2
501–1000 birr ($27-55)	117	26
1001–3000 birr ($56-166)	176	39.2
3001–5000 birr ($167-277)	11	2.4
>5000 birr ($ > 277)	1	0.2

*$* dollar.

### Sexual history related to HIV/AIDS and intention to use condom

Among the respondents, it was found that 122 (27.1%) had sex in the past one year, of whom 93 (76.2%) were males and 29 (23.8%) were females. From these, 36 (29.5%) had only one sexual partner, 71(58.2%) had two sexual partners and 15(12.3%) of them had more than two sexual partners in the last 12 months. The mean age of sexual debut was 16.2 years. Of those who had sex in the past one year 45(37%) never used condom, 12(9.8%) used condom sometimes and 65(53.2%) used condom every time. The main reasons for not using condom during sexual intercourse was attributed to have trust in one partner 23(19%), opposition from sexual partner 17(14%), religious disapproval 14(11%), dislike to use 11(9%) and unavailability of condoms 7(6%). Only 150 (33.3%) of study subjects have intention to use condom in the next sexual encounter, the majority 300(66.7%) have no intention to use condom in the next sexual encounter (Table 2).

**Table 2:** Past sexual behaviour and intention to use condom at next sexual activity among students in Agena preparatory school, Guraghe zone, Ethiopia, 2011

**Variable**	**Frequency**	**Percent (%)**
Had sex in the past one year		
Yes	122	27.1
No	328	72.9
Age of sex started		
Mean (average age)	16.2 (S.D =0.97)	
**Number of sexual partner in the past one year**		
1	36	29.5
2	71	58.2
≥3	15	12.3
Condom utilization in the past one year		
Yes	77	63.1
No	45	36.9
Frequency of condom use (n = 122)		
Never	45	37
Sometime	12	9.8
Every time	65	53.2
Intention to use condom (n = 450)		
Yes	150	33.3
No	300	66.7
Reason for not using condom		
Trust in one partner	23	19
Opposition from partner	17	14
Religious disapproval	14	11
Dislike to use	11	9
Unavailability of condom	7	6

*S.D* standard deviation.

### Knowledge on transmission and prevention of HIV/AIDS and misconceptions

About 420 (93.3%) subjects believed that the mode of transmission is unprotected sex, 374 (77.1%) through infected instruments and 231(51.3%) from pregnant mother to child. Almost similar proportion of the respondents, 381(84.7%) and 377(83.8%) perceived that being faithful to one partner and abstinence are preventive methods of HIV/AIDS respectively. About 321 (71.3%) of the respondents perceived that correct and consistent use of condom is a preventive method of HIV/AIDS. With regards to misconceptions related to HIV/AIDS, few study subjects replied that HIV is transmissible via mosquito’s bite 40(8.9) and 39 (8.7%) of the respondents said that HIV can be transmitted by eating with PLHIVs and 18(4%) by eggs of a hen that had swallowed a used condom (Table 3).

**Table 3:** Knowledge related to transmission and prevention of HIV/AIDS, and Misconceptions among students in Agena preparatory school, Guraghe zone, Ethiopia, 2011

**Variable**	**Frequency**	**Percent (%)**
Knowledge of modes of transmission of HIV/AIDS		
Unprotected sex	420	93.3
Infected instruments	374	77.1
MTCT	231	51.3
Unsafe blood transfusions	327	72.7
Knowledge of preventive methods of HIV/AIDS		
Being faithful to one partner	381	84.7
Abstinence	377	83.8
Correct and consistent use of condom	321	71.3
Misconceptions about transmission of HIV infection		
Mosquito’s bites	40	8.9
Eating with PLWHA	39	8.7
Eating eggs of a hen swallowed used condom	18	4

### Perceived susceptibility

Student’s attitude towards perceiving themselves as susceptible to HIV infection was assessed and the result indicated that more than half of them had low perception of acquiring the infection. The mean score was 2.69.

### Perceived severity

More than half of the respondents had low perception towards severity of HIV/AIDS. The mean score for perceived severity was 1.85.

### Perceived benefit

More than half of the respondents agreed that condom use was low to prevent HIV/AIDS. The mean score for the perceived benefit of condom use was 1.27.

### Perceived barrier

The majority of the students had high level of perception on barrier for condom use. The mean score was 3.82.

### Self-efficacy

Half of the respondents had high confidence in using condom. The mean score for self-efficacy were 2.78 (Table 4).

**Table 4:** Mean and standard deviation of theoretical variable of Agena preparatory school students, Guraghe zone, Ethiopia, 2011

**Theoretical variable**	**Mean (0–5)**	**Standard deviation**
Perceived susceptibility	2.69	1.14
Perceived severity	1.85	0.75
Perceived benefit	1.27	0.17
Perceived barrier	3.82	1.69
Self-efficacy	2.78	1.94

Bivariate analysis showed the following variables have statistical association with intention to use condom. Male were more likely to have intention to use condom than female, [COR = 2.37 95% CI (1.54, 3.66)]. Those who had high perception on susceptibility were less likely to have intention to use condom as compared to those having low perception on susceptibility [COR = 0.59 95% CI (0.4, 0.89)]. Students who had high self-efficacy were more likely to have intention to use condom than those having low self efficacy [COR = 29.0 95% CI (15.3, 55.2)]. Those students who have high perceived barrier were more likely to have intention to use condom [COR = 2.28 95% CI (1.53-3.4)] than those who have low perceived barrier (Table 5).

**Table 5:** Relationship of the theoretical constructs with intention to use condom among students of Agena preparatory school, Guraghe zone, Ethiopia, 2011

**Variable**	**Intention to use condom**		**No intention to use condom**		**COR (Crude Odd Ratio) 95% CI**
Age					
15–19	141 (94%)		269	(89.7%)	1.8 (0.83–3.89)
20–25	9 (6%)		31 (10.3%)		1
Sex					
Male	122 (81%)		166	(55.3%)	2.37 (1.54–3.66)
Female	38	(19%)	134	(44.7%)	1
Educational status					
Grade 11	79	(52.7%)	137	(45.7%)	1
Grade 12	71 (47.3%)		163	(54.3%)	0.75 (0.51–1.19)
Perceived					
Susceptibility					
Low	93	(62%)	148	(49.3)	1
High	57	(38%)	152	(50.7%)	0.59 (0.4–0.89)*
Perceived					
Severity					
Low	89	(59.3%)	150	(50%)	1
High	61	(40.7%)	150	(50%)	0.68 (0.46–1)
Perceived benefit					
Low	86	(57.3%)	161	(53.7%)	1
High	64	(42.7%)	139	(42.3%)	0.86 (0.58–1.3)
Perceived barrier					
Low	91	(60.7%)	121	(80.7%)	1
High	59	(39.3%)	179	(19.3%)	2.28 (1.53–3.4)**
Self efficacy					
Low	12	(8%)	215	(71.7%)	1
High	138 (92%)		85 (29.3%)		29 (15.3–55)**

** = p-value <0.0001 * = p-value < 0.05.

After adjusting for the other variables the multivariate analysis revealed that those who have high perceived susceptibility to HIV/AIDS were more likely to have intention to use condom [AOR = 1.94 95% CI (1.16-3.2)] compared to their counter parts and those who have high self efficacy have more likely to have intention to use condom [AOR = 27 95% CI (14.4-54.2)] than others (Table 6).

**Table 6:** Odds ratios from logistic regression models predicting intention to use condom among students in Agena preparatory school, Guraghe zone, Ethiopia, 2011

**Variable**	**Intention to use condom**		**No intention to use condom**	**COR (Crude Odd Ratio) 95% CI**	**AOR (Adjusted Odd Ratio) 95% CI**
Sex					
Male	122 (81%)		166 (55.3%)	2.37 (1.54–3.66)	1.31 (0.76–2.36)
Female	38	(19%)	134 (44.7%)	1	1
Educational					
Status					
Grade 11	79	(52.7%)	137 (45.7%)	1	1
Grade 12	71	(47.3%)	163 (54.3%)	0.75 (0.51–1.19)	1.41 (0.85–2.34)
Perceived					
Susceptibility					
Low	93	(62%)	148 (49.3%)	1	1
High	57	(38%)	152 (50.7%)	0.59 (0.4–0.89)	1.94 (1.16–3.2)*
Perceived					
Barrier					
Low	91	(60.7%)	121 (80.7%)	1	1
High	59	(39.3%)	179 (19.3%)	2.28 (1.53–3.4)	1.53 (0.92–2.54)
Self efficacy					
Low	12	(8%)	215 (71.7%)	1	1
High	138 (92%)		85 (29.3%)	29 (15.3–55)	27 (14.4–54.2)**

** = p-value <0.0001 * = p-value < 0.05.

## Discussion

HIV/AIDS is known to be the major challenges of developing countries. However it is much more challenging in sub-Saharan Africa affecting particularly the productive segments of their population. Thus, it was in line with this fact that a cross sectional study done in Agena preparatory school students concerning their current risky sexual behaviours and condom use intention using health behaviour model. The study investigated different factors such as socio-demographic characteristics and psychosocial factors in relation to their effect on the use of condom and condom use intention.

One fourth of the study participants had sexual exposure and it was found to be higher among males (76.2 Vs 23.8%). The prevalence of condom use (73.1%) among those who have sexual intercourse in the past one year was higher than the study done at Gondar College of Medical Sciences (37.1%) and Agaro high school (54.4%) which may be explained by the study time difference and the effect of continued information dissemination through different mechanisms [7,8].

AIDS risk behaviours such as having contact with multiple sexual partners and having unprotected sex (none use of condom) was common in this study. Among those 122 (27.1%) who have sex in the past one year in this study most participants 86(70.5%) have two or more sexual partners. Moreover, out of those who had sex in the past one year 45(36.9%) never used condom. Some studies on HIV-risk behaviours showed that despite adequate knowledge about HIV/AIDS higher proportion of people especially youths continue to experiment with high risk behaviours (the behaviours known to place individuals at risk of HIV infection) and having multiple sexual partners which is the key concern in much of the sub-Saharan Africa. History of multiple sexual partners was the main risk factor for HIV infection in Ethiopia [10].

The average age of sexual debut was 16.2 years which put them at higher risk of acquiring HIV infection since those students with age of less than 18 years were with higher likelihood of lower intention to use condom in their next sexual encounter. Similar finding was observed in other studies [8].

The current study findings were consistent with the theory of health belief model where perceived susceptibility and self-efficacy had the major predictive power than the other predictor variables in predicting intention to use condom at the next sexual intercourse. Similar findings on intention to use condom, but from a different analyses approach has also been documented among Tanzanian adolescents. Furthermore, Among Nigerian University students and Sera lion Freetown University Students, Concerning the relative importance of theoretical components, the strong effect was observed from perceived susceptibility and self efficacy in predicting the intention to use condom at the next sexual intercourse [11].

Human behaviours that predispose people to acquiring and transmitting HIV were assessed. The key benefits from this finding that involved both high and low risk groups of population is to fill the gaps of information existing in behavioural survey in Ethiopia in general and emerging regions in particular. Uganda and Thailand used behavioural survey as an evidence to prove the reduction of AIDS prevalence in the past couples of years as a result of change in high-risk human behaviours gained earlier [10,12,13]. For effective prevention and handling of the epidemic, definitive and concrete knowledge on means of viral transmission and rejecting prevalent misconception are crucial. Assessment of high-risk behaviours in Ethiopia was initiated as early as 1990s. High score especially in some mode of viral transmission was documented which showed some success especially in raising awareness both in the general and sub group of population [14].

Adequate knowledge on transmission of HIV by itself is of no use if individuals don’t know that they can get infection from asymptomatic carrier who looks well and healthy, had low level of awareness and condom use, which is quite comparable to many studies in Ethiopia and Africa [15]. Furthermore if the respondents had little awareness that asymptomatic healthy carriers could transmit the disease, they may not refrain themselves from engaging in sexual relation with this peoples. Those factors may result in a big challenge in an effort to fight against the disease.

Finally, the implication of the study findings is clear that the significant associations which emerged from this research should be incorporated into AIDS risk reduction programs. Individuals should be made aware of the dangers associated with being less concerned about multiple partnership and use condom in different situations. Programs must be designed in such a way that relevant others would also practice safe sex. Using relevant others for passing message and skills is required. Students will change their behaviours in response to the true expectations of others more than through changes in individual beliefs or attitudes. This suggests that deficit of shifting social norms, individuals won't change. Therefore, the intervention plan must ensure that all channels reaching the network contain the same message to increase the perception that the new practice has wide support. In addition, intervention, which encourages community discussion on the issue, can be included with the assumption that such discussion will accelerate the process of social norm diffusion.

This study has some limitations. Firstly it is expected to be prone for the limitation of cross-sectional survey (temporal relationship). Secondly the explicit wisdom, values or culture of a given ethnic group, religion or previous environment are expected to have some kind of influence on current decisions related to sexual behaviour in general, condom use status in particular. Thirdly it may be exposed to social desirability bias. Lastly one of the predictor variables ‘self efficacy’ had a larger AOR and a wider confidence interval resulting in low precision to the true value.

## Conclusions

Intention to use condom in the next sexual encounter is very low despite having a relatively acceptable level of knowledge on mode of HIV/AIDs transmission and prevention. It was found to be associated with perceived susceptibility and self-efficacy. Multiples sexual partner relation was common among those who had sex in the past one year. And significant number of the study participants have never used condom during sexual intercourse.

Therefore the following are the possible actions; Information Education and Communication (IEC) on reducing number of sexual partners along with condom use promotion using behavioural change communication strategies focusing on increasing youths perceived susceptibility to HIV/AIDS and self efficacy should be the primary strategy of HIV/AIDS prevention process in target population of the study area and the wider youth community in Ethiopia. With this, emphasis has also to be given towards avoiding other high-risk behaviours. Further investigation concerning the socio-psychological and cultural factors should be performed to achieve the intended positive behavioural changes pertaining to HIV/AIDS prevention effort particularly adolescents of Ethiopia.

## Competing interests

The authors would like to declare that we have no competing interest in this particular study.

## Authors’ contributions

GG has conceived of the study, carried out the overall design and execution of the study, performed data collection and statistical analysis. ND has participated in the revision of the design of the study, data collection techniques and helped the statistical analysis. GT participated in the drafting of the manuscript and assisted the design of the study and data analysis. All authors read and finally approved this manuscript for submission.

## Authors’ information

Negussie Deyessa and Gezahegn Tesfaye are co-authors.
